# Supplementary material for: Emollient prescribing formularies in England and Wales: a cross-sectional study
Source: BMJ Open. 2018 Jun 12;8(6):e022009. doi: 10.1136/bmjopen-2018-022009 (PMC6009537; doi:10.1136/bmjopen-2018-022009)
Supplement: Supplementary data [file bmjopen-2018-022009-SP1.pdf]

Supplementary tables

Table S1: Names of emollients named in formularies in alphabetical order, by formulation (n=109 emollients)

Creams	Ointments	Lotions	Gels	Sprays	Other
n = 56 (51%)	n = 17 (16%)	n = 12 (11%)	n = 8 (7%)	n = 3 (3%)	n = 13 (12%)
AproDerm Cream	Bepanthen	Aveeno Lotion	AproDerm Gel	Dermamist Spray	Arachis oil
AproDerm Colloidal Oat Cream	Cetraben Ointment	Aveeno Lotion with lavender	Doublebase Gel	Emollin	Aripro foam
Aquadrate Cream	Diprobase Ointment	Calamine	Doublebase Dayleve Gel	Evolve plus (Lanolin emollient spray)	ClearZal
Aquamol Cream	Emulsifying ointment BP	Cetraben Lotion	Isomol Gel		Coconut Oil BPC
Aquamax Cream	Epaderm Ointment	Dermol 500 Lotion	Generic Isopropyl myristate 15%/Liquid paraffin 15% gel		Dermatonics Heel Balm
Aqueous Cream	Fifty:50 Ointment (White soft/Liquid paraffin 50/50)	Diprobase Lotion	MyriBase Gel		Flexitol Heel Balm
Arjun Cream - all menthol percentages	Hydrous Ointment	E45 Lotion	Oilatum Gel		Keri
Aveeno Cream	Hydromol Ointment	Eucerin Intensive Lotion	Zerodouble Gel		Olive oil

Aveeno Intensive Relief Hand Cream	Paraffin Liquid soft	Eucerin Lotion			Skin kind deep skin cleanser
Balneum Cream	Paraffin White soft	Oilatum Lotion			Skin kind moisturiser plus
Balneum Plus Cream	Paraffin Yellow soft	QV Lotion			Skin kind moisturiser
Calmurid Cream	White soft/Liquid paraffin 50/50	Vaseline Dermacare Lotion			Skin kind unique deep skin moisturiser
Cetomacrogol Cream Formula A 1989	Pure Health (White soft/Liquid paraffin 50/50)				Sunflower oil
Cetraben Cream	QV Ointment				
Decubal clinic Cream	30:30 Ointment				
Dermacool (menthol in aqueous cream)	Zeroderm Ointment				
Dermol Cream	Zinc Ointment BP				
Diprobase Cream					
Drapolene					
Dexeryl Cream					
E45 Cream					
E45 itch relief					
Eczmol Cream					
Epaderm Cream					

Epimax Cream					
Eucerin Cream					
Eucerin Intensive Cream					
Eucerin 5% dry skin relief Cream					
Eumocream					
Eurax Cream					
Exocream					
Flexitol 10% cream					
Hewletts					
Hydromol Cream					
Hydromol Intensive					
Imuderm					
Kamillosan					
Linola Gamma					
Lipobase					
Neutrogena Dermatological Cream					
Nutraplus Cream					
Oilatum Cream					
Oilatum Junior Cream					
QV Cream					
Skin salvation cream					

Soffen Cream					
Ultrabase Cream					
Unguentum M Cream					
Urea Cream 10%					
Urea 5% Special Preparation					
ZeroAQS Cream					
Zerobase Cream					
Zerocream					
Zeroguent Cream					
Zinc Cream BP					
Zinc and Castor oil Cream					

Table S2: Top five emollients recommended by type (n=102)

Lotion			Cream			Gel			Ointment		
**Name**	**n**	**%**	**Name**	**n**	**%**	**Name**	**n**	**%**	**Name**	**n**	**%**
Dermol 500 Lotion	71	70	Cetraben Cream	70	69	Doublebase Gel	62	61	White soft/Liquid paraffin 50/50	81	79
E45 Lotion	31	30	Zerobase Cream	62	61	Zerodouble Gel	39	38	Emulsifying Ointment BP	80	78
Cetraben Lotion	30	29	Dermol Cream	60	59	Doublebase Dayleve Gel	21	21	Hydromol Ointment	72	71
Aveeno Lotion	28	27	Zerocream	56	55	Oilatum Gel	8	8	Zeroderm Ointment	59	58
Eucerin Intensive Lotion	22	22	Balneum plus cream	53	52	Isomol Gel	6	6	Paraffin White Soft	41	40

Table S3: Top five recommended emollients by order of formulary preference (n=102 formularies)

Emollient	Number (%) of formularies recommending as				Recommended without ranking
	First line	Second line	Third line	Fourth line	
Emulsifying Ointment BP	31 (30%)	8 (8%)	0 (0%)	0 (0%)	41 (40%)
Zeroderm Ointment	28 (27%)	11 (11%)	1 (1%)	1 (1%)	18 (18%)
Zerobase Cream	27 (26%)	8 (8%)	2 (2%)	0 (0%)	25 (25%)
Zerocream	25 (25%)	5 (5%)	1 (1%)	0 (0%)	25 (25%)
White soft/ Liquid paraffin 50/50	23 (23%)	9 (9%)	2 (2%)	0 (0%)	48 (47%)
