# Supplementary material for: Emollient prescribing formularies in England and Wales: a cross-sectional study
Source: BMJ Open. 2018 Jun 12;8(6):e022009. doi: 10.1136/bmjopen-2018-022009 (PMC6009537; doi:10.1136/bmjopen-2018-022009)
Supplement: Author's manuscript [file bmjopen-2018-022009.draft_revisions.pdf]

BMJ Open

BMJ Open is committed to open peer review. As part of this commitment we make the peer review history of every article we publish publicly available.

When an article is published we post the peer reviewers' comments and the authors' responses online. We also post the versions of the paper that were used during peer review. These are the versions that the peer review comments apply to.

The versions of the paper that follow are the versions that were submitted during the peer review process. They are not the versions of record or the final published versions. They should not be cited or distributed as the published version of this manuscript.

BMJ Open is an open access journal and the full, final, typeset and author-corrected version of record of the manuscript is available on our site with no access controls, subscription charges or pay-per-view fees (http://bmjopen.bmj.com).

If you have any questions on BMJ Open's open peer review process please email info.bmjopen@bmj.com

BMJ Open

Emollient prescribing formularies in England and Wales: cross-sectional study

Journal:	*BMJ Open*
Manuscript ID	bmjopen-2018-022009
Article Type:	Research
Date Submitted by the Author:	29-Jan-2018
Complete List of Authors:	Chan, Jonathan; University of Bristol, Population Health Sciences<br>Boyd, Grace; University of Bristol, Population Health Sciences<br>Quinn, Patrick; University of Bristol, Population Health Sciences<br>Ridd, Matthew; University of Bristol, Population Health Sciences
Keywords:	Eczema < DERMATOLOGY, PRIMARY CARE, Protocols & guidelines < HEALTH SERVICES ADMINISTRATION & MANAGEMENT

SCHOLARONE™
Manuscripts

For peer review only

Title page

Title

Emollient prescribing formularies in England and Wales: cross-sectional study

Authors

Jonathan P Chan[1], Grace Boyd[1], Patrick A Quinn[1], Matthew J Ridd[1]

[1] Population Health Sciences, Bristol Medical School, University of Bristol, Canynge Hall, 39 Whatley Road, Bristol BS8 2PS

Corresponding author

Dr Matthew J Ridd, Canynge Hall, 39 Whatley Road, Bristol BS8 2PS, m.ridd@bristol.ac.uk

Manuscript word, table and figure count

Words: 1924

Tables: 3 (+3 supplementary tables)

Figures: 1

Keywords

Atopic eczema, prescribing formularies, primary healthcare

Copyright

The Corresponding Author has the right to grant on behalf of all authors and does grant on behalf of all authors, a worldwide licence to the Publishers and its licensees in perpetuity, in all forms, formats and media (whether known now or created in the future), to i) publish, reproduce, distribute, display and store the Contribution, ii) translate the Contribution into other languages, create adaptations, reprints, include within collections and create summaries, extracts and/or, abstracts of the Contribution, iii) create any other derivative work(s) based on the Contribution, iv) to exploit all subsidiary rights in the Contribution, v) the inclusion of electronic links from the Contribution to third party material where-ever it may be located; and, vi) licence any third party to do any or all of the above.

For peer review only

Abstract

Objective: To identify and compare emollient formularies across all Clinical Commissioning Groups (CCGs) and Local Health Boards (LHBs) in England and Wales.

Design: Formularies were retrieved via CCG/LHB websites or Google search (October 2016-February 2017). Data on structure and content were extracted and descriptive analyses undertaken.

Setting: 209 English CCGs and 7 Welsh LHBs.

Main outcome measures: Number and structure of formularies; number, type and name of emollients and bath additive recommended & not recommended, and any rationale given.

Results: 102 formularies were identified, which named 109 emollients and 24 bath additives. Most were structured in an "order of preference" (63%) and/or formulation (51%) format. Creams and ointments were the most commonly recommended types of emollients and three ointments were the most commonly recommended specific emollients (71-79% of formularies). However, there was poor consensus over which emollient should be used first-line and four out of ten of the most recommended lotions and creams contained antimicrobials or urea. Patient preference (60%) and/or cost (58%) were the most common reasons given for the recommendations. Of the 82% of formularies that recommend the use of bath additives, 75% did not give any reasons for their recommendation.

Conclusions: Emollient formularies in England and Wales vary widely in their structure, recommendations and rationale. The reasons for such inconsistencies are unclear, risk confusion and make for inequitable regional variation. There is poor justification for multiple different, conflicting formularies.

Word count: 232/300

Strengths and limitations of this study

- This is the first study to compare all emollient formularies in England and Wales.
- The findings highlight the hitherto undescribed number of different emollient formularies and the unexplained variation in format and recommendations – including contradictory advice.
- Due to the labour-intensive nature of data extraction and constant change in CCG/LHB service configuration and formularies, it is impossible to have an up-to-date national picture at any one point in time.
- Very few formularies referred to specific diseases or populations (age, ethnicity), so we were unable report recommendations at this level.
- Ambiguity in many of the formularies, differences in researcher interpretation, and transcription errors may mean inaccuracies were introduced during the data extraction process.

For peer review only

Introduction

The annual spend on emollients in England alone in 2015/16 was £116.2 million.[1] Despite their accepted value in dry skin conditions such as atopic eczema, there are multiple products which come in different formulations (ointments, gels, creams, lotions, sprays and bath additives) and a weak evidence base to guide their use.[2-4]

Most prescribing of emollients happens in primary care and Clinical Commissioning Groups (CCGs) in England and Local Health Boards (LHBs) in Wales, who have the responsibility for the provision of NHS care on their locality, maintain local prescribing formularies. NICE guidelines states that such information should be published "… in a clear, simple and transparent way, so that patients, the public and stakeholders can easily understand it."[5]

The need for multiple formularies is questionable, although the number, type and content of recommendations made has never been examined. Therefore, we sought to identify and compare the structure and content of emollient formularies across England and Wales, including any rationale behind the recommendations given.

Methods

Data collection

First, CCGs in England and LHBs in Wales were identified using the NHS choices (www.nhs.uk) & NHS England websites (www.england.nhs.uk, as listed in October 2016) and the NHS Wales website (www.wales.nhs.uk, February 2017). All available formularies were identified by systematically searching each CCG/LHB website or undertaking Google searches using relevant keywords.

JPC and GB extracted data from each formulary into "leave on" and bath additive Excel® spreadsheets templates respectively. These templates were initially piloted and developed with a sample of formularies. To ensure consistency of data extraction, a codebook detailing the various coding options was developed alongside. An independent check of 10 formularies identified discrepancies in 4.7% (57/1220) of "leave on" emollient (PAQ) and 9.8% (44/448) of bath additive (JPC) data points.

Analysis

Data were then imported and analysed descriptively using STATA (v14). All percentages were rounded to the nearest whole number unless stated otherwise.

Patient involvement

"Which emollient is the most effective and safe in treating eczema" was identified as a priority for research in a patient and clinician-led prioritisation exercise published in 2013.6 Public and patient involvement in subsequent eczema research led by MJR has reinforced the importance of understanding the variation in emollient prescribing and recommendations made by different doctors and specialist nurses better.[7-9] However, patients were not involved in the design of the study or the interpretation of the findings.

For peer review only

Patients were not involved in design, conduct or analysis during this study.

Results

89% (185/209) of CCGs had an emollient formulary and all seven LHBs had an emollient formulary (Fig. 1). 71% (131/185) CCGs shared an emollient formulary with at least one other CCG, but all 7 LHBs had their own individual emollient formulary. Some CCGs (24/209) withheld formularies for internal use only or did not respond, even after direct correspondence, meaning data could only be collected from 89% of CCGs. Therefore, overall 102 formularies were examined.

The structure of formularies varied but for emollients most (63%, 64/102) adopted a "rank" (traffic light, medal or number) structure, with by formulation (51%, 52/102) being next most common, followed by basic (alphabetical) list (47%, 48/102) and by skin dryness (10%, 10/102) (categories not mutually exclusive). 34% (35/102) of formularies gave the specific costs for each emollient.

Emollients

Over 109 different emollients were named, with 93 of these recommended by at least one formulary. Almost every formulary recommended at least one cream (99%) or ointment (98%) (see Table 1). Most formularies also recommended at least one lotion or gel (both 85%), but only 29% recommending a spray. No formularies actively recommended against use of creams, lotions, ointments or gels. However, 9% of formularies did not recommend sprays and most formularies (62%) made no mention of them. 'Other' emollients were most commonly (58%) not recommend by formularies. This category contained more unusual products such as food based oils and balms (Table S1).

Cream and ointments were the most commonly recommended types of emollients (table 2), with 99% and 98% of formularies recommending at least one product from each group respectively. Of specifically recommended emollients, three of the "top five" are ointments (Table 2). However, between 2% and 9% of formularies specifically did not recommend one or more of these products. When ranked by emollient type (lotion, cream, gel and ointment), three products which contain urea or antimicrobials (Dermol 500 Lotion, Dermol Cream, Balneum plus Cream) appear in the "top five" (table S2). Among the 64 formularies that specified an order of preference for recommended emollients, generic emulsifying ointment was the most popular first-line emollient, followed by three "Zero" products and white soft/liquid paraffin 50/50 (table S3). Aqueous cream was the most commonly not recommended emollient (45% of formularies, Table 2), although again there was disagreement between formularies, with up to 16% recommending one or more of the top five emollients not recommended.

The most common reasons given for the recommendations made were patient preference (60%, 61/102) and/or product cost (58%, 59/102). 42% (43/102) of formularies gave other reasons, the most common being "the greasier the emollient, the better the effect". Other rationale given for not recommending emollients included concerns over food based ingredients and the ability of active ingredients to penetrate the skin. No rationale was given in 28% (29/102).

For peer review only

Bath additives

Of the 82% (84/102) of formularies that recommended the use of bath additives, 75% (64/84) did not give any rationale. Six formularies noted that evidence to support their use was lacking, eight recommended their use only in specific circumstances and six cited "possible benefit for some patients".

There was no mention of bath additives in 7% (7/102) of formularies and 11% (11/102) did not recommend their routine use. 4% (4/102) stated that patients should buy their own. The rational to justify not prescribing bath emollients was variable, the most common (non-exclusive) reasons cited were: lack of evidence (5/11), concerns about safety (4/11), the potential to under-treat eczema by distracting from leave-on emollient use (3/11) and cost (1/11). Three formularies did not give a reason.

Most formularies (92%, 77/84) presented their recommendations as individually named bath additives. A small number of formularies (8%, 7/84) also suggested that any emollient (except for "50:50") could be used in the bath instead of a specific bath additive. Regarding the use of bath additives with antimicrobials, they were recommended in 74% (62/84) of formularies, not mentioned in 25% (21/84), and one advised against their use ever. Of those recommending their use, 34% (21/62) specified only "in the presence of infection".

Overall 19 regular and five antimicrobial containing bath additives were named (see table 3). No bath emollients were specifically listed as not recommended. The five most commonly recommended bath emollients were Oilatum, Hydromol, Dermol 600, Oilatum plus and Balneum plus. Some formularies restricted use of specific bath additives, for example Aveeno was often only recommended if paraffin intolerant or following no response to other bath emollients.

Discussion

We found that most CCGs and LHBs (192/216) had a formulary, with 102 unique emollient formularies between them. Of the 109 different emollients named, creams and ointments were the most commonly recommended types and three ointments (White soft/Liquid paraffin 50/50, Emulsifying ointment and Hydromol ointment) the most frequently specified products. However, there was poor consensus over which emollient should be used first-line and four out of ten of the most recommended lotions and cream contained antimicrobials or urea. Patient preference (60%) and/or cost (58%) were the most commonly reasons given for the recommendations. However, cost recommendations were on a "price per gram or millilitre" basis, rather than proper cost-effectiveness evidence. 11% of formularies did not recommend their use of bath additives, while the 82% that did named 24 different bath additives and most (75%) did not give any reasons for their recommendation.

This is the first study to compare all emollient formularies in England and Wales. Formularies were identified, and data extracted, in a systematic way. Ambiguity in many of the formularies, differences in interpretation and transcription errors may mean inaccuracies were introduced during this process. In the time between collecting and reporting the data, two further CCGs have merged (new total 207) and a rapid review of the emollient formularies in November 2017 identified that 50% have been updated. However, most changes appear to be minor and the key findings of the

For peer review only

number of formularies and similarities/differences between the different formularies hold true. Because of the labour-intensive nature of data extraction, the constant change in NHS service configuration, and CCGs/LHBs independently maintaining >100 formularies, it is impossible to have an up-to-date national picture at any one point in time. Very few formularies referred to specific diseases or populations (age, ethnicity), so we were unable report recommendations at this level.

One significant factor contributing to the lack of agreement between the formularies is the weak evidence base. Several recent systematic reviews have all concluded that good quality research comparing the effectiveness or acceptability of emollients is very limited.[2-4] Likewise, there is an absence of evidence to demonstrate the clinical and cost effectiveness of bath additives.[3] The greatest consensus is around recommendations for ointments, one appeal being their low cost, although current NICE guidance on atopic eczema in children (from 2007) notes that they are often less acceptable and are therefore "not usually suitable as a first-line treatment".[10] The predominance of emollients which contain urea or antimicrobials is surprising: these products are usually second-line treatments, generally cost more than standard emollients, and are more likely to irritate skin. It is encouraging to see 45% of formularies actively not recommending aqueous cream, probably reflecting the 2013 MHRA safety report on adverse skin reactions in children with eczema.[11] There is a disparity between formulary recommendations and recent community dispensing data from NHS England[12 13] where the top five dispensed emollients were Cetraben cream, Doublebase gel, Diprobase cream, Aveeno cream and Dermol 500 lotion. This may be due to a lag between formulary implementation and prescribing patterns changing, wilful ignorance of formularies on the part of prescribers or strong patient preference for alternative emollients.

We know that much emollient prescribing is done on a "trial and error" basis,[14 15] and it is unclear the extent to which all these different formularies help reduce (or indeed contribute) to this problem. While local formularies can reflect the needs of needs of the population served, the justification in the case of emollients/bath additives is weak (and indeed local factors contributing to decision making not specified in any of the formularies). Furthermore, we cannot understand why the formularies of 24 CCGs are not publicly available. In addition to the time and cost of different medicine management teams and area prescribing committees developing individual emollient formularies, differences between them serves to cause confusion to prescribers and patients, especially when they move between areas. This combined with the discrepancy between emollient dispensing data and formulary recommendations suggests the purpose of emollient formularies should be rethought and/or communicated more effectively.

While the "correct" emollient is the "one that the child will use",[10] is a pragmatic approach to emollients prescribing, it is not one that is supported by formulary recommendations that have a weak evidence base. Future research should further explore the disparity between recommended and observed prescribing of emollients, including changes over time in formulary recommendations and prescribing patterns. The results of on-going pragmatic trials such as Best Emollient for Eczema (BEE, comparing different emollient types) and Bath Additives for the Treatment of Childhood Eczema (BATHE, bath additives in addition to usual care) will provide clinical and cost-effectiveness data which will inform, and hopefully unify, formulary recommendations to the benefit of patients and clinicians.

For peer review only

References

1. Wilson L, Smith K. Cost effective prescribing of emollients. PrescQIPP: East Anglia Medicines Information Service, 2015.
2. Van Zuuren EJ, Fedorowicz Z, Christensen R, et al. Emollients and moisturisers for eczema. *Cochrane Database of Systematic Reviews* 2017(2) doi: 10.1002/14651858.CD012119.pub2.
3. Nankervis H, Thomas KS, Delamere FM, et al. What is the evidence base for atopic eczema treatments? A summary of published randomized controlled trials. *British Journal of Dermatology* 2017;176(4):910-27. doi: 10.1111/bjd.14999
4. Jacobi A, Mayer A, Augustin M. Keratolytics and Emollients and Their Role in the Therapy of Psoriasis: a Systematic Review. *Dermatology and Therapy* 2015;5(1):1-18. doi: 10.1007/s13555-015-0068-3
5. NICE. Developing and updating local formularies. NICE, 2014. [Available from: https://www.nice.org.uk/guidance/mpg1, accessed 15/01/18]
6. Batchelor JM, Ridd MJ, Clarke T, et al. The Eczema Priority Setting Partnership: a collaboration between patients, carers, clinicians and researchers to identify and prioritize important research questions for the treatment of eczema. *British Journal of Dermatology* 2013;168(3):577-82. doi: 10.1111/bjd.12040
7. Ridd MJ, Garfield K, Gaunt DM, et al. Choice of Moisturiser for Eczema Treatment (COMET): feasibility study of a randomised controlled parallel group trial in children recruited from primary care. *BMJ Open* 2016;6(11) doi: 10.1136/bmjopen-2016-012021
8. Ridd M, Redmond N, Hollinghurst S, et al. Choice of Moisturiser for Eczema Treatment (COMET): study protocol for a randomized controlled trial. *Trials* 2015;16(1):304. doi: 10.1186/s13063-015-0830-y
9. Powell K, Le Roux E, Banks JP, et al. Developing a written action plan for children with eczema: a qualitative study. *British Journal of General Practice* 2018;68(667):e81-e89. doi: 10.3399/bjgp17X693617
10. NICE. NICE clinical guideline CG057 Atopic eczema in under 12s: diagnosis and management: NICE; 2007 [Available from: https://www.nice.org.uk/guidance/cg57 accessed 08/11/16.
11. MHRA. Aqueous cream: contains sodium lauryl sulfate which may cause skin reactions, particularly in children with eczema: Medicines and Healthcare products Regulatory Agency (MHRA); 2013 [updated 11/02/2016. Available from: http://www.mhra.gov.uk/safety-public-assessment-reports/CON251956 accessed 07/01/2017.
12. NHSBSA. Prescription Cost Analysis (PCA) data 2017 [Available from: http://www.nhsbsa.nhs.uk/PrescriptionServices/3494.aspx accessed 27 March 2017.
13. NHS-Wales. Primary Care Services - Prescription Cost Analysis 2017 [Available from: http://www.primarycareservices.wales.nhs.uk/prescription-cost-analysis accessed 16/04/17.
14. Le Roux E, Powell K, Banks J, et al. GPs' experience of diagnosing and managing eczema in children: qualitative study. *BJGP* 2017
15. Santer M, Muller I, Yardley L, et al. Parents' and carers' views about emollients for childhood eczema: qualitative interview study. *BMJ Open* 2016;6(8) doi: 10.1136/bmjopen-2016-011887

For peer review only

Footnotes

Acknowledgments

The authors gratefully acknowledge Ms Daisy Gaunt for her advice on utilising the software STATA and to Ms Faye Shelton for reviewing the emollient formularies in November 2017.

Author contribution

MJR conceived the study. JPC and GB undertook data extraction and analyses (for emollients and bath additives respectively), supervised by MJR. Independent data scrutiny was done by PQ (emollients) and JPC (bath additives). JPC, GB & MJR interpreted the data. JPC drafted the manuscript and MJR substantially revised it, with contributions from GB and PQ.

All authors had full access to all the data in the study and take responsibility for the integrity of the data and the accuracy of the data analysis. MJR is guarantor for the study.

Funding

MJR was supported by the National Institute for Health Research during this study (NIHR Post Doctoral Fellowship, PDF-2014-07-013). JPC and GB received funding from the Academy of Medical Sciences INSPIRE scheme, University of Bristol. The views expressed in this article are those of the author(s) and not necessarily those of the NHS, the NIHR, or the Department of Health. Funders of this study had no role in the study design; collection, analysis, and interpretation of the data; writing of the manuscript; or decision to submit the manuscript for publication. The authors are independent from funders.

Conflicts of interests

Dr Ridd reports grants from National Institute for Health Research (NIHR) Post Doctoral Fellowship (PDF-2014-07-013) and NIHR HTA (11/153/01 and 15/130/07). Mr Chan and Ms Boyd report grants from The Academy of Medical Sciences INSPIRE scheme. Mr Quinn has nothing to disclose. Funders of this study had no role in the study design; collection, analysis, and interpretation of the data; writing of the manuscript; or decision to submit the manuscript for publication. The authors are independent from funders.

Ethics

This study did not require ethics approval.

Data sharing

Full dataset and technical appendix available from the corresponding author.

For peer review only

Transparency

The lead author (JPC) affirms that the manuscript is an honest, accurate, and transparent account of the study being reported; that no important aspects of the study have been omitted; and that any discrepancies from the study as planned have been explained.

For peer review only

Tables

Table 1: Formularies' recommendations by type of emollient (n=102)

Formulation	Recommended		Not recommended		Not specified	
	n	%	n	%	n	%
Lotion	87	85	0	0	15	15
Cream	101	99	0	0	1	1
Gel	87	85	0	0	15	15
Ointment	100	98	0	0	2	2
Spray	30	29	9	9	63	62
Other	43	42	59	58	0	0

For peer review only

Table 2: Top five emollients most commonly recommended and not recommended by formularies (all types) (n=102)

	Number (%) of formularies			
Name of emollient	Recommending		Not recommending	
	n	%	n	%
**Top five recommended**				
White soft/Liquid paraffin 50/50	81	79	2	2
Emulsifying Ointment BP	80	78	2	2
Hydromol Ointment	72	71	6	6
Dermol 500 Lotion	71	70	9	9
Cetraben Cream	70	69	6	6
**Top five not recommended**				
Aqueous Cream	11	11	46	45
E45 itch relief	10	10	22	22
Ultrabase Cream	16	16	20	20
Nutraplus Cream	14	14	19	19
Dermamist Spray	12	12	18	18
Lipobase	3	3	18	18

Table 3: Regular and antimicrobial containing bath emollients recommended by formularies (n=84)

	Recommended		Not recommended	
	n	%	n	%
**Regular bath emollients**				
Oilatum	51	61	14	17
Hydromol	43	51	10	12
Balneum plus	32	38	9	11
Balneum	30	36	17	20
Aveeno	24	29	20	24
Zeroneum	24	29	20	24
Dermalo	22	26	20	24
Zerolatum	20	24	21	25
Double base	19	23	12	14
Cetraben	18	21	16	19
QV bath oil	15	18	20	24
LPL 63.4	13	15	10	12
E45	13	15	21	25
Aqueous cream BP	11	13	9	11
Diprobath	10	12	18	21
Oilatum Junior	8	10	9	11
Zerolatum plus	4	5	16	19
ZeroAQS	2	2	7	8
Zerozole	1	1	18	21
**Bath emollients containing antimicrobial agent**				
Dermol 600	41	49	8	10
Oilatum plus	34	40	11	13
Emulsiderm	22	26	18	21
Dermol	14	17	14	17
Dermol 500	4	5	7	8

For peer review only

Figure 1: Flowchart of the Clinical Commissioning Groups (CCGs)/Local Health Boards (LHBs) in England/Wales and number of available emollient formularies

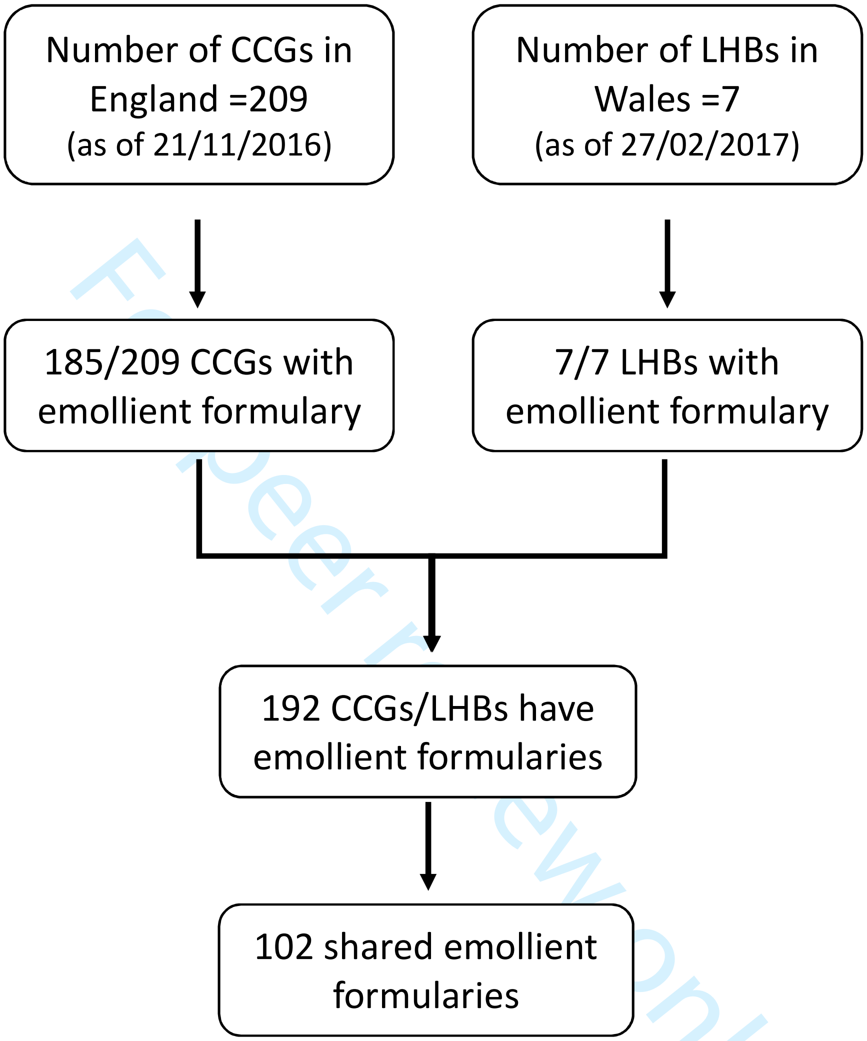

For peer review only - http://bmjopen.bmj.com/site/about/guidelines.xhtml

For peer review only

For peer review only - http://bmjopen.bmj.com/site/about/guidelines.xhtml

Supplementary tables

Table S1: Names of emollients named in formularies in alphabetical order, by formulation (n=109 emollients)

Creams	Ointments	Lotions	Gels	Sprays	Other
n = 56 (51%)	n = 17 (16%)	n = 12 (11%)	n = 8 (7%)	n = 3 (3%)	n = 13 (12%)
AproDerm Cream	Bepanthen	Aveeno Lotion	AproDerm Gel	Dermamist Spray	Arachis oil
AproDerm Colloidal Oat Cream	Cetraben Ointment	Aveeno Lotion with lavender	Doublebase Gel	Emollin	Aripro foam
Aquadrate Cream	Diprobase Ointment	Calamine	Doublebase Dayleve Gel	Evolve plus (Lanolin emollient spray)	ClearZal
Aquamol Cream	Emulsifying ointment BP	Cetraben Lotion	Isomol Gel		Coconut Oil BPC
Aquamax Cream	Epaderm Ointment	Dermol 500 Lotion	Generic Isopropyl myristate 15%/Liquid paraffin 15% gel		Dermatonics Heel Balm
Aqueous Cream	Fifty:50 Ointment (White soft/Liquid paraffin 50/50)	Diprobase Lotion	MyriBase Gel		Flexitol Heel Balm
Arjun Cream - all menthol percentages	Hydrous Ointment	E45 Lotion	Oilatum Gel		Keri
Aveeno Cream	Hydromol Ointment	Eucerin Intensive Lotion	Zerodouble Gel		Olive oil

For peer review only - http://bmjopen.bmj.com/site/about/guidelines.xhtml

Aveeno Intensive Relief Hand Cream	Paraffin Liquid soft	Eucerin Lotion			Skin kind deep skin cleanser
Balneum Cream	Paraffin White soft	Oilatum Lotion			Skin kind moisturiser plus
Balneum Plus Cream	Paraffin Yellow soft	QV Lotion			Skin kind moisturiser
Calmurid Cream	White soft/Liquid paraffin 50/50	Vaseline Dermacare Lotion			Skin kind unique deep skin moisturiser
Cetomacrogol Cream Formula A 1989	Pure Health (White soft/Liquid paraffin 50/50)				Sunflower oil
Cetraben Cream	QV Ointment				
Decubal clinic Cream	30:30 Ointment				
Dermacool (menthol in aqueous cream)	Zeroderm Ointment				
Dermol Cream	Zinc Ointment BP				
Diprobase Cream					
Drapolene					
Dexeryl Cream					
E45 Cream					
E45 itch relief					
Eczmol Cream					
Epaderm Cream					

For peer review only - http://bmjopen.bmj.com/site/about/guidelines.xhtml

Epimax Cream					
Eucerin Cream					
Eucerin Intensive Cream					
Eucerin 5% dry skin relief Cream					
Eumocream					
Eurax Cream					
Exocream					
Flexitol 10% cream					
Hewletts					
Hydromol Cream					
Hydromol Intensive					
Imuderm					
Kamillosan					
Linola Gamma					
Lipobase					
Neutrogena Dermatological Cream					
Nutraplus Cream					
Oilatum Cream					
Oilatum Junior Cream					
QV Cream					
Skin salvation cream					

For peer review only

For peer review only - http://bmjopen.bmj.com/site/about/guidelines.xhtml

Soffen Cream					
Ultrabase Cream					
Unguentum M Cream					
Urea Cream 10%					
Urea 5% Special Preparation					
ZeroAQS Cream					
Zerobase Cream					
Zerocream					
Zeroguent Cream					
Zinc Cream BP					
Zinc and Castor oil Cream					

For peer review only

For peer review only - http://bmjopen.bmj.com/site/about/guidelines.xhtml

Table S2: Top five emollients recommended by type (n=102)

Lotion			Cream			Gel			Ointment		
**Name**	**n**	**%**	**Name**	**n**	**%**	**Name**	**n**	**%**	**Name**	**n**	**%**
Dermol 500 Lotion	71	70	Cetraben Cream	70	69	Doublebase Gel	62	61	White soft/Liquid paraffin 50/50	81	79
E45 Lotion	31	30	Zerobase Cream	62	61	Zerodouble Gel	39	38	Emulsifying Ointment BP	80	78
Cetraben Lotion	30	29	Dermol Cream	60	59	Doublebase Dayleve Gel	21	21	Hydromol Ointment	72	71
Aveeno Lotion	28	27	Zerocream	56	55	Oilatum Gel	8	8	Zeroderm Ointment	59	58
Eucerin Intensive Lotion	22	22	Balneum plus cream	53	52	Isomol Gel	6	6	Paraffin White Soft	41	40

For peer review only

Table S31: Top five recommended emollients by order of formulary preference (n=102 formularies)

Emollient	Number (%) of formularies recommending as				Recommended without ranking
	First line	Second line	Third line	Fourth line	
Emulsifying Ointment BP	31 (30%)	8 (8%)	0 (0%)	0 (0%)	41 (40%)
Zeroderm Ointment	28 (27%)	11 (11%)	1 (1%)	1 (1%)	18 (18%)
Zerobase Cream	27 (26%)	8 (8%)	2 (2%)	0 (0%)	25 (25%)
Zerocream	25 (25%)	5 (5%)	1 (1%)	0 (0%)	25 (25%)
White soft/ Liquid paraffin 50/50	23 (23%)	9 (9%)	2 (2%)	0 (0%)	48 (47%)

For peer review only

STROBE 2007 (v4) Statement—Checklist of items that should be included in reports of *cross-sectional studies*

Section/Topic	Item #	Recommendation	Reported on page #
**Title and abstract**	1	(*a*) Indicate the study's design with a commonly used term in the title or the abstract	1
		(*b*) Provide in the abstract an informative and balanced summary of what was done and what was found	2
**Introduction**			
Background/rationale	2	Explain the scientific background and rationale for the investigation being reported	3
Objectives	3	State specific objectives, including any prespecified hypotheses	2 & 3
**Methods**			
Study design	4	Present key elements of study design early in the paper	3
Setting	5	Describe the setting, locations, and relevant dates, including periods of recruitment, exposure, follow-up, and data collection	3
Participants	6	(*a*) Give the eligibility criteria, and the sources and methods of selection ~~of participants~~	3
Variables	7	Clearly define all outcomes, ~~exposures, predictors, potential confounders, and effect modifiers. Give diagnostic criteria, if applicable~~	2 & 3
Data sources/ measurement	8*	For each variable of interest, give sources of data and details of methods of assessment (measurement). ~~Describe comparability of assessment methods if there is more than one group~~	3
Bias	9	Describe any efforts to address potential sources of bias	*3*
Study size	10	Explain how the study size was arrived at	N/A
Quantitative variables	11	Explain how quantitative variables were handled in the analyses. If applicable, describe which groupings were chosen and why	3
Statistical methods	12	(*a*) Describe all statistical methods, including those used to control for confounding	3
		(*b*) Describe any methods used to examine subgroups and interactions	N/A
		(*c*) Explain how missing data were addressed	3 & 6
		(*d*) If applicable, describe analytical methods taking account of sampling strategy	N/A
		(*e*) Describe any sensitivity analyses	N/A
**Results**			

For peer review only

Participants	13*	(a) Report numbers of individuals at each stage of study—eg numbers potentially eligible, examined for eligibility, confirmed eligible, included in the study, completing follow-up, and analysed	3 & 12
		(b) Give reasons for non-participation at each stage	6
		(c) Consider use of a flow diagram	12
Descriptive data	14*	(a) Give characteristics of study participants (eg demographic, clinical, social) and information on exposures and potential confounders	N/A
		(b) Indicate number of participants with missing data for each variable of interest	N/A
Outcome data	15*	Report numbers of outcome events or summary measures	3, 4, 5
Main results	16	(*a*) Give unadjusted estimates and, if applicable, confounder-adjusted estimates and their precision (eg, 95% confidence interval). Make clear which confounders were adjusted for and why they were included	3, 4, 5
		(*b*) Report category boundaries when continuous variables were categorized	N/A
		(*c*) If relevant, consider translating estimates of relative risk into absolute risk for a meaningful time period	N/A
Other analyses	17	Report other analyses done—eg analyses of subgroups and interactions, and sensitivity analyses	N/A
**Discussion**			
Key results	18	Summarise key results with reference to study objectives	5
Limitations	19	Discuss limitations of the study, taking into account sources of potential bias or imprecision. Discuss both direction and magnitude of any potential bias	2 & 5
Interpretation	20	Give a cautious overall interpretation of results considering objectives, limitations, multiplicity of analyses, results from similar studies, and other relevant evidence	5 & 6
Generalisability	21	Discuss the generalisability (external validity) of the study results	5 & 6
**Other information**			
Funding	22	Give the source of funding and the role of the funders for the present study and, if applicable, for the original study on which the present article is based	8

*Give information separately for cases and controls in case-control studies and, if applicable, for exposed and unexposed groups in cohort and cross-sectional studies.

Note: An Explanation and Elaboration article discusses each checklist item and gives methodological background and published examples of transparent reporting. The STROBE checklist is best used in conjunction with this article (freely available on the Web sites of PLoS Medicine at http://www.plosmedicine.org/, Annals of Internal Medicine at http://www.annals.org/, and Epidemiology at http://www.epidem.com/). Information on the STROBE Initiative is available at www.strobe-statement.org.

For peer review only

BMJ Open

Emollient prescribing formularies in England and Wales: cross-sectional study

Journal:	*BMJ Open*
Manuscript ID	bmjopen-2018-022009.R1
Article Type:	Research
Date Submitted by the Author:	22-Mar-2018
Complete List of Authors:	Chan, Jonathan; University of Bristol, Population Health Sciences<br>Boyd, Grace; University of Bristol, Population Health Sciences<br>Quinn, Patrick; University of Bristol, Population Health Sciences<br>Ridd, Matthew; University of Bristol, Population Health Sciences
**Primary Subject Heading**:	Dermatology
Secondary Subject Heading:	General practice / Family practice, Pharmacology and therapeutics
Keywords:	Eczema < DERMATOLOGY, PRIMARY CARE, Protocols & guidelines < HEALTH SERVICES ADMINISTRATION & MANAGEMENT

SCHOLARONE™
Manuscripts

For peer review only - http://bmjopen.bmj.com/site/about/guidelines.xhtml

Title page

Title

Emollient prescribing formularies in England and Wales: cross-sectional study

Authors

Jonathan P Chan[1], Grace Boyd[1], Patrick A Quinn[1], Matthew J Ridd[1]

[1] Population Health Sciences, Bristol Medical School, University of Bristol, Canynge Hall, 39 Whatley Road, Bristol BS8 2PS

Corresponding author

Dr Matthew J Ridd, Canynge Hall, 39 Whatley Road, Bristol BS8 2PS, m.ridd@bristol.ac.uk

Manuscript word, table and figure count

Words: 1924

Tables: 3 (+3 supplementary tables)

Figures: 1

Keywords

Atopic eczema, prescribing formularies, primary healthcare

Copyright

The Corresponding Author has the right to grant on behalf of all authors and does grant on behalf of all authors, a worldwide licence to the Publishers and its licensees in perpetuity, in all forms, formats and media (whether known now or created in the future), to i) publish, reproduce, distribute, display and store the Contribution, ii) translate the Contribution into other languages, create adaptations, reprints, include within collections and create summaries, extracts and/or, abstracts of the Contribution, iii) create any other derivative work(s) based on the Contribution, iv) to exploit all subsidiary rights in the Contribution, v) the inclusion of electronic links from the Contribution to third party material where-ever it may be located; and, vi) licence any third party to do any or all of the above.

For peer review only

Abstract

Objective: To identify and compare emollient formularies across all Clinical Commissioning Groups (CCGs) and Local Health Boards (LHBs) in England and Wales.

Design: Formularies were retrieved via CCG/LHB websites or Google search (October 2016-February 2017). Data on structure and content were extracted and descriptive analyses undertaken.

Setting: 209 English CCGs and 7 Welsh LHBs.

Main outcome measures: Number and structure of formularies; number, type and name of emollients and bath additive recommended & not recommended, and any rationale given.

Results: 102 formularies were identified, which named 109 emollients and 24 bath additives. Most were structured in an “order of preference” (63%) and/or formulation (51%) format. Creams and ointments were the most commonly recommended types of emollients and three ointments were the most commonly recommended specific emollients (71-79% of formularies). However, there was poor consensus over which emollient should be used first-line and four out of ten of the most recommended lotions and creams contained antimicrobials or urea. Patient preference (60%) and/or cost (58%) were the most common reasons given for the recommendations. Of the 82% of formularies that recommend the use of bath additives, 75% did not give any reasons for their recommendation.

Conclusions: Emollient formularies in England and Wales vary widely in their structure, recommendations and rationale. The reasons for such inconsistencies are unclear, risk confusion and make for inequitable regional variation. There is poor justification for multiple different, conflicting formularies.

Word count: 232/300

Strengths and limitations of this study

- This is the first study to compare in a systematic way all of the emollient formularies in England and Wales.
- Due to the labour-intensive nature of data extraction and constant change in CCG/LHB service configuration and formularies, it is impossible to have an up-to-date national picture at any one point in time.
- Very few formularies referred to specific diseases or populations (age, ethnicity), so we were unable report recommendations at this level.
- Ambiguity in many of the formularies, differences in researcher interpretation, and transcription errors may mean inaccuracies were introduced during the data extraction process.

For peer review only

Introduction

The annual spend on emollients in England alone in 2015/16 was £116.2 million.[1] Despite their accepted value in dry skin conditions such as atopic eczema, there are multiple products which come in different formulations (ointments, gels, creams, lotions, sprays and bath additives) and a weak evidence base to guide their use.[2-4]

Most prescribing of emollients happens in primary care and Clinical Commissioning Groups (CCGs) in England and Local Health Boards (LHBs) in Wales, who have the responsibility for the provision of NHS care on their locality, maintain local prescribing formularies. NICE guidelines states that such information should be published "… in a clear, simple and transparent way, so that patients, the public and stakeholders can easily understand it."[5]

The need for multiple formularies is questionable, although the number, type and content of recommendations made has never been examined. Therefore, we sought to identify and compare the structure and content of emollient formularies across England and Wales, including any rationale behind the recommendations given.

Methods

Data collection

First, CCGs in England and LHBs in Wales were identified using the NHS choices (www.nhs.uk), NHS England (www.england.nhs.uk, as listed in October 2016) and NHS Wales websites (www.wales.nhs.uk, February 2017). All available formularies were identified by systematically searching each CCG/LHB website or undertaking Google searches using relevant keywords.

JPC and GB extracted data from each formulary into "leave on" and bath additive Excel® spreadsheets templates respectively. These templates were initially piloted and developed with a sample of formularies. To ensure consistency of data extraction, a codebook detailing the various coding options was developed alongside. An independent check of 10 formularies identified discrepancies in 4.7% (57/1220) of "leave on" emollient (PAQ) and 9.8% (44/448) of bath additive (JPC) data points. Where disagreements arose, the two data extractors discussed and agreed what should be recorded; most stemmed from differing interpretations of vague and/or inconsistent formularies, rather than random errors.

Analysis

Data were then imported and analysed descriptively using STATA (v14). All percentages were rounded to the nearest whole number unless stated otherwise.

Patient involvement

"Which emollient is the most effective and safe in treating eczema?" was identified as a priority for research in a patient and clinician-led prioritisation exercise published in 2013.[6] Public and patient involvement in subsequent eczema research led by MJR has reinforced the importance of understanding the variation in emollient prescribing and recommendations made by different

For peer review only

doctors and specialist nurses better.[7-9] However, patients were not involved in the design of the study or the interpretation of the findings.

Results

89% (185/209) of CCGs had an emollient formulary and all seven LHBs had an emollient formulary (Fig. 1). 71% (131/185) CCGs shared an emollient formulary with at least one other CCG, but all 7 LHBs had their own individual emollient formulary. Some CCGs (24/209) withheld formularies for internal use only or did not respond, even after direct correspondence, meaning data could only be collected from 89% of CCGs. Therefore, overall 102 formularies were examined.

The structure of formularies varied but for emollients most (63%, 64/102) adopted a "rank" (traffic light, medal or number) structure, with by formulation (51%, 52/102) being next most common, followed by basic (alphabetical) list (47%, 48/102) and by skin dryness (10%, 10/102) (categories not mutually exclusive). 34% (35/102) of formularies gave the specific costs for each emollient.

Emollients

Over 109 different emollients were named, with 93 of these recommended by at least one formulary. Almost every formulary recommended at least one cream (99%) or ointment (98%) (see Table 1). Most formularies also recommended at least one lotion or gel (both 85%), but only 29% recommended a spray. No formularies censuredthe use of creams, lotions, ointments or gels. However, 9% of formularies recommended against sprays and most formularies (62%) made no mention of them. 'Other' types of emollients were most commonly (58%) not recommend by formularies. This category contained more unusual products such as food-based oils and balms (Table S1).

Cream and ointments were the most commonly recommended types of emollients (Table 2), with 99% and 98% of formularies recommending at least one product from each group respectively. Of specifically recommended and named emollients, three of the "top five" are ointments (Table 2). However, between 2% and 9% of formularies specifically did not recommend one or more of these products. When ranked by emollient type (lotion, cream, gel and ointment), three products which contain urea or antimicrobials (Dermol 500 Lotion, Dermol Cream, Balneum plus Cream) appear in the "top five" (table S2). Among the 64 formularies that specified an order of preference for recommended emollients, generic emulsifying ointment was the most popular first-line emollient, followed by three "Zero" products and white soft/liquid paraffin 50/50 (Table S3). Aqueous cream was the most common emollient not recommended (45% of formularies, Table 2), although again there was disagreement between formularies, with up to 16% recommending one or more of the top five not recommended emollients.

The most common reasons given for the recommendations made were patient preference (60%, 61/102) and/or product cost (58%, 59/102). 42% (43/102) of formularies gave other reasons, the most common being "the greasier the emollient, the better the effect". Other rationale given for not recommending emollients included concerns over food-based ingredients and the ability of active ingredients to penetrate the skin. No rationale was given in 28% (29/102).

For peer review only

Bath additives

Of the 82% (84/102) of formularies that recommended the use of bath additives, 75% (64/84) did not give any rationale. Six formularies noted that evidence to support their use was lacking, eight recommended their use only in specific circumstances and six cited "possible benefit for some patients".

There was no mention of bath additives in 7% (7/102) of formularies and 11% (11/102) did not recommend their routine use. 4% (4/102) stated that patients should buy their own. The rational for not prescribing bath emollients was variable, the most common (non-exclusive) reasons cited were: lack of evidence (5/11), concerns about safety (4/11), the potential to under-treat eczema by distracting from leave-on emollient use (3/11) and cost (1/11). Three formularies did not give a reason.

Most formularies (92%, 77/84) presented their recommendations as individually named bath additives. A small number of formularies (8%, 7/84) also suggested that any emollient (except for "50:50") could be used in the bath instead of a specific bath additive. Regarding the use of bath additives with antimicrobials, they were recommended in 74% (62/84) of formularies, not mentioned in 25% (21/84), and one advised against their use ever. Of those recommending their use, 34% (21/62) specified "only in the presence of infection".

Overall 19 regular and five antimicrobial containing bath additives were named (see table 3). The five most commonly recommended bath emollients were Oilatum, Hydromol, Dermol 600, Oilatum plus and Balneum plus. Some formularies restricted use of specific bath additives, for example Aveeno was often only recommended if paraffin intolerant or following no response to other bath emollients.

Discussion

We found that most CCGs and LHBs (192/216) had a formulary, with 102 unique emollient formularies between them. Of the 109 different emollients named, creams and ointments were the most commonly recommended types and three ointments (White soft/Liquid paraffin 50/50, Emulsifying ointment and Hydromol ointment) the most frequently specified products. However, there was poor consensus over which emollient should be used first-line and four out of ten of the most recommended lotions and cream contain antimicrobials or urea. Patient preference (60%) and/or cost (58%) were the most common reasons given for the recommendations. However, cost recommendations were on a "price per gram or millilitre" basis, rather than proper cost-effectiveness evidence. 11% of formularies did not recommend their use of bath additives, while the 82% that did named 24 different bath additives and most (75%) did not give any reasons for their recommendation.

This is the first study to compare all emollient formularies in England and Wales. Formularies were identified, and data extracted, in a systematic way. Ambiguity in many of the formularies, differences in interpretation and transcription errors may mean inaccuracies were introduced during this process. In the time between collecting and reporting the data, two further CCGs have merged (new total 207) and a rapid review of the emollient formularies in November 2017 identified that 50% have been updated. However, most changes appear to be minor and the key findings of the

For peer review only

number of formularies and similarities/differences between the different formularies hold true. Because of the labour-intensive nature of data extraction, the constant change in NHS service configuration, and CCGs/LHBs independently maintaining >100 formularies, it is impossible to have an up-to-date national picture at any one point in time. Very few formularies referred to specific diseases or populations (age, ethnicity), so we were unable report recommendations at this level.

One significant factor contributing to the lack of agreement between the formularies is the weak evidence base. Several recent systematic reviews have all concluded that good quality research comparing the effectiveness or acceptability of emollients is very limited.[2-4] Likewise, there is an absence of evidence to demonstrate the clinical and cost effectiveness of bath additives.[3] The greatest consensus is around recommendations for ointments, one appeal being their low cost, although current NICE guidance on atopic eczema in children (from 2007) notes that they are often less acceptable and are therefore "not usually suitable as a first-line treatment".[10] The predominance of emollients which contain urea or antimicrobials is surprising: these products are usually second-line treatments, generally cost more than standard emollients, and are more likely to irritate skin.[1 11] It is encouraging to see 45% of formularies actively not recommending aqueous cream, probably reflecting the 2013 MHRA safety report on adverse skin reactions in children with eczema.[12] There is a disparity between formulary recommendations and recent NHS community dispensing data (for children and adults)[13 14] where the top five dispensed emollients were Cetraben cream, Doublebase gel, Diprobase cream, Aveeno cream and Dermol 500 lotion. There may be many reasons for this observation, beyond the scope of this paper, but which may include: a time lag between formulary implementation and prescribing patterns changing; prescriber lack of familiarity with formulary recommendations; and/or strong clinician or patient preference for non-recommended emollients.

We know that much emollient prescribing is done on a "trial and error" basis,[15 16] but it is unclear the extent to which all these different formularies help reduce (or indeed contribute) to this problem. While local formularies can reflect the needs of the population served, the justification in the case of emollients/bath additives is weak (and indeed local factors contributing to decision making not specified in any of the formularies). Furthermore, we cannot understand why the formularies of 24 CCGs are not publicly available. In addition to the time and cost of different medicine management teams and area prescribing committees developing individual emollient formularies, differences between them serves to cause confusion to prescribers and patients, especially when they move between areas. This combined with the discrepancy between emollient dispensing data and formulary recommendations suggests the purpose of emollient formularies should be rethought and/or communicated more effectively.

While the "correct" emollient is the "one that the child will use",[10] is a pragmatic approach to emollient prescribing, it is not one that is supported by formulary recommendations that have a weak evidence base. Future research should further explore the disparity between recommended and observed prescribing of emollients, including changes over time in formulary recommendations and prescribing patterns. The results of on-going pragmatic trials such as Best Emollient for Eczema (BEE, comparing different emollient types) and Bath Additives for the Treatment of Childhood Eczema (BATHE, bath additives in addition to usual care)[17] will provide clinical and cost-effectiveness data which will inform, and hopefully simplify, formulary recommendations to the benefit of patients and clinicians.

For peer review only

References

1. Wilson L, Smith K. Cost effective prescribing of emollients. PrescQIPP: East Anglia Medicines Information Service, 2015.
2. Van Zuuren EJ, Fedorowicz Z, Christensen R, et al. Emollients and moisturisers for eczema. *Cochrane Database of Systematic Reviews* 2017(2) doi: 10.1002/14651858.CD012119.pub2.
3. Nankervis H, Thomas KS, Delamere FM, et al. What is the evidence base for atopic eczema treatments? A summary of published randomized controlled trials. *British Journal of Dermatology* 2017;176(4):910-27. doi: 10.1111/bjd.14999
4. Jacobi A, Mayer A, Augustin M. Keratolytics and Emollients and Their Role in the Therapy of Psoriasis: a Systematic Review. *Dermatology and Therapy* 2015;5(1):1-18. doi: 10.1007/s13555-015-0068-3
5. NICE. Developing and updating local formularies. NICE, 2014. [Available from: https://www.nice.org.uk/guidance/mpg1, accessed 15/01/18]
6. Batchelor JM, Ridd MJ, Clarke T, et al. The Eczema Priority Setting Partnership: a collaboration between patients, carers, clinicians and researchers to identify and prioritize important research questions for the treatment of eczema. *British Journal of Dermatology* 2013;168(3):577-82. doi: 10.1111/bjd.12040
7. Ridd MJ, Garfield K, Gaunt DM, et al. Choice of Moisturiser for Eczema Treatment (COMET): feasibility study of a randomised controlled parallel group trial in children recruited from primary care. *BMJ Open* 2016;6(11) doi: 10.1136/bmjopen-2016-012021
8. Ridd M, Redmond N, Hollinghurst S, et al. Choice of Moisturiser for Eczema Treatment (COMET): study protocol for a randomized controlled trial. *Trials* 2015;16(1):304. doi: 10.1186/s13063-015-0830-y
9. Powell K, Le Roux E, Banks JP, et al. Developing a written action plan for children with eczema: a qualitative study. *British Journal of General Practice* 2018;68(667):e81-e89. doi: 10.3399/bjgp17X693617
10. NICE. NICE clinical guideline CG057 Atopic eczema in under 12s: diagnosis and management: NICE; 2007 [Available from: https://www.nice.org.uk/guidance/cg57 accessed 08/11/16.
11. Ling TC, Highet AS. Irritant reactions to an antiseptic bath emollient. *Journal of Dermatological Treatment* 2000;11(4):263-67. doi: 10.1080/09546630050517216
12. MHRA. Aqueous cream: contains sodium lauryl sulfate which may cause skin reactions, particularly in children with eczema: Medicines and Healthcare products Regulatory Agency (MHRA); 2013 [updated 11/02/2016. Available from: http://www.mhra.gov.uk/safety-public-assessment-reports/CON251956 accessed 07/01/2017.
13. NHSBSA. Prescription Cost Analysis (PCA) data 2017 [Available from: http://www.nhsbsa.nhs.uk/PrescriptionServices/3494.aspx accessed 27 March 2017.
14. NHS-Wales. Primary Care Services - Prescription Cost Analysis 2017 [Available from: http://www.primarycareservices.wales.nhs.uk/prescription-cost-analysis accessed 16/04/17.
15. Le Roux E, Powell K, Banks J, et al. GPs' experience of diagnosing and managing eczema in children: qualitative study. BJGP 2018; 68: e73-e80. DOI: 10.3399/bjgp18X694529

For peer review only

16. Santer M, Muller I, Yardley L, et al. Parents' and carers' views about emollients for childhood eczema: qualitative interview study. *BMJ Open* 2016;6(8) doi: 10.1136/bmjopen-2016-011887

17. Santer M, Rumsby K, Ridd MJ, et al. Bath additives for the treatment of childhood eczema (BATHE): protocol for multicentre parallel group randomised trial. *BMJ Open* 2015;5(10) DOI: 10.1136/bmjopen-2015-009575

Footnotes

Acknowledgments

The authors gratefully acknowledge Ms Daisy Gaunt for her advice on utilising the software STATA and to Ms Faye Shelton for reviewing the emollient formularies in November 2017.

Author contribution

MJR conceived the study. JPC and GB undertook data extraction and analyses (for emollients and bath additives respectively), supervised by MJR. Independent data scrutiny was done by PQ (emollients) and JPC (bath additives). JPC, GB & MJR interpreted the data. JPC drafted the manuscript and MJR substantially revised it, with contributions from GB and PQ.

All authors had full access to all the data in the study and take responsibility for the integrity of the data and the accuracy of the data analysis. MJR is guarantor for the study.

Funding

MJR was supported by the National Institute for Health Research during this study (NIHR Post Doctoral Fellowship, PDF-2014-07-013). JPC and GB received funding from the Academy of Medical Sciences INSPIRE scheme, University of Bristol. The views expressed in this article are those of the author(s) and not necessarily those of the NHS, the NIHR, or the Department of Health. Funders of this study had no role in the study design; collection, analysis, and interpretation of the data; writing of the manuscript; or decision to submit the manuscript for publication. The authors are independent from funders.

Conflicts of interests

Dr Ridd reports grants from National Institute for Health Research (NIHR) Post Doctoral Fellowship (PDF-2014-07-013) and NIHR HTA (11/153/01 and 15/130/07). Mr Chan and Ms Boyd report grants from The Academy of Medical Sciences INSPIRE scheme. Mr Quinn has nothing to disclose. Funders of this study had no role in the study design; collection, analysis, and interpretation of the data; writing of the manuscript; or decision to submit the manuscript for publication. The authors are independent from funders.

Ethics

This study did not require ethics approval.

For peer review only

Data sharing

Full dataset and technical appendix have been uploaded as supplementary files.

Transparency

The lead author (JPC) affirms that the manuscript is an honest, accurate, and transparent account of the study being reported; that no important aspects of the study have been omitted; and that any discrepancies from the study as planned have been explained.

For peer review only

Figure and Tables

Figure 1: Flowchart of the Clinical Commissioning Groups (CCGs)/Local Health Boards (LHBs) in England/Wales and number of available emollient formularies

[See accompanying document]

Table 1: Formularies' recommendations by type of emollient (n=102)

Formulation	Recommended		Not recommended		Neither recommended nor not recommended	
	n	%	n	%	n	%
Lotion	87	85	0	0	15	15
Cream	101	99	0	0	1	1
Gel	87	85	0	0	15	15
Ointment	100	98	0	0	2	2
Spray	30	29	9	9	63	62
Other	43	42	59	58	0	0

For peer review only

Table 2: Top five emollients most commonly recommended and not recommended by formularies (all types) (n=102)

	Number (%) of formularies			
**Name of emollient**	**Recommending**		**Not recommending**	
	n	%	n	%
**Top five recommended**				
White soft/Liquid paraffin 50/50	81	79	2	2
Emulsifying Ointment BP	80	78	2	2
Hydromol Ointment	72	71	6	6
Dermol 500 Lotion	71	70	9	9
Cetraben Cream	70	69	6	6
**Top five not recommended**				
Aqueous Cream	11	11	46	45
E45 itch relief	10	10	22	22
Ultrabase Cream	16	16	20	20
Nutraplus Cream	14	14	19	19
Dermamist Spray	12	12	18	18
Lipobase	3	3	18	18

Table 3: Regular and antimicrobial containing bath emollients recommended by formularies (n=84)

	Recommended		Not recommended	
	n	%	n	%
**Regular bath emollients**				
Oilatum	51	61	14	17
Hydromol	43	51	10	12
Balneum plus	32	38	9	11
Balneum	30	36	17	20
Aveeno	24	29	20	24
Zeroneum	24	29	20	24
Dermalo	22	26	20	24
Zerolatum	20	24	21	25
Double base	19	23	12	14
Cetraben	18	21	16	19
QV bath oil	15	18	20	24
LPL 63.4	13	15	10	12
E45	13	15	21	25
Aqueous cream BP	11	13	9	11
Diprobath	10	12	18	21
Oilatum Junior	8	10	9	11
Zerolatum plus	4	5	16	19
ZeroAQS	2	2	7	8
Zerozole	1	1	18	21
**Bath emollients containing antimicrobial agent**				
Dermol 600	41	49	8	10
Oilatum plus	34	40	11	13

For peer review only

Emulsiderm	22	26	18	21
Dermol	14	17	14	17
Dermol 500	4	5	7	8

For peer review only

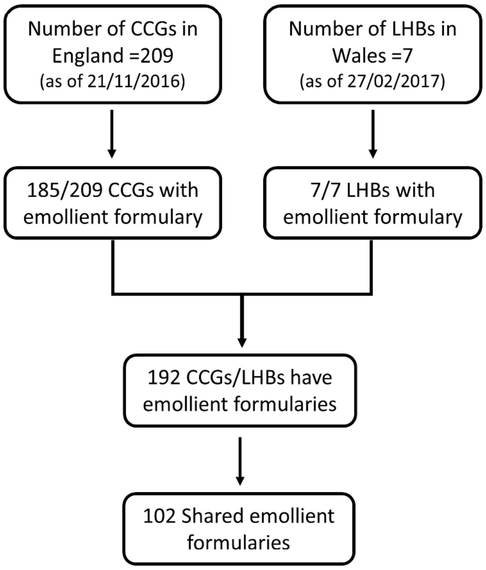

Figure 1: Flowchart of the Clinical Commissioning Groups (CCGs)/Local Health Boards (LHBs) in England/Wales and number of available emollient formularies

254x190mm (300 x 300 DPI)

For peer review only - http://bmjopen.bmj.com/site/about/guidelines.xhtml

Supplementary tables

Table S1: Names of emollients named in formularies in alphabetical order, by formulation (n=109 emollients)

Creams	Ointments	Lotions	Gels	Sprays	Other
n = 56 (51%)	n = 17 (16%)	n = 12 (11%)	n = 8 (7%)	n = 3 (3%)	n = 13 (12%)
AproDerm Cream	Bepanthen	Aveeno Lotion	AproDerm Gel	Dermamist Spray	Arachis oil
AproDerm Colloidal Oat Cream	Cetraben Ointment	Aveeno Lotion with lavender	Doublebase Gel	Emollin	Aripro foam
Aquadrate Cream	Diprobase Ointment	Calamine	Doublebase Dayleve Gel	Evolve plus (Lanolin emollient spray)	ClearZal
Aquamol Cream	Emulsifying ointment BP	Cetraben Lotion	Isomol Gel		Coconut Oil BPC
Aquamax Cream	Epaderm Ointment	Dermol 500 Lotion	Generic Isopropyl myristate 15%/Liquid paraffin 15% gel		Dermatonics Heel Balm
Aqueous Cream	Fifty:50 Ointment (White soft/Liquid paraffin 50/50)	Diprobase Lotion	MyriBase Gel		Flexitol Heel Balm
Arjun Cream - all menthol percentages	Hydrous Ointment	E45 Lotion	Oilatum Gel		Keri
Aveeno Cream	Hydromol Ointment	Eucerin Intensive Lotion	Zerodouble Gel		Olive oil

For peer review only - http://bmjopen.bmj.com/site/about/guidelines.xhtml

Aveeno Intensive Relief Hand Cream	Paraffin Liquid soft	Eucerin Lotion			Skin kind deep skin cleanser
Balneum Cream	Paraffin White soft	Oilatum Lotion			Skin kind moisturiser plus
Balneum Plus Cream	Paraffin Yellow soft	QV Lotion			Skin kind moisturiser
Calmurid Cream	White soft/Liquid paraffin 50/50	Vaseline Dermacare Lotion			Skin kind unique deep skin moisturiser
Cetomacrogol Cream Formula A 1989	Pure Health (White soft/Liquid paraffin 50/50)				Sunflower oil
Cetraben Cream	QV Ointment				
Decubal clinic Cream	30:30 Ointment				
Dermacool (menthol in aqueous cream)	Zeroderm Ointment				
Dermol Cream	Zinc Ointment BP				
Diprobase Cream					
Drapolene					
Dexeryl Cream					
E45 Cream					
E45 itch relief					
Eczmol Cream					
Epaderm Cream					

For peer review only

Epimax Cream					
Eucerin Cream					
Eucerin Intensive Cream					
Eucerin 5% dry skin relief Cream					
Eumocream					
Eurax Cream					
Exocream					
Flexitol 10% cream					
Hewletts					
Hydromol Cream					
Hydromol Intensive					
Imuderm					
Kamillosan					
Linola Gamma					
Lipobase					
Neutrogena Dermatological Cream					
Nutraplus Cream					
Oilatum Cream					
Oilatum Junior Cream					
QV Cream					
Skin salvation cream					

For peer review only

For peer review only - http://bmjopen.bmj.com/site/about/guidelines.xhtml

Soffen Cream					
Ultrabase Cream					
Unguentum M Cream					
Urea Cream 10%					
Urea 5% Special Preparation					
ZeroAQS Cream					
Zerobase Cream					
Zerocream					
Zeroguent Cream					
Zinc Cream BP					
Zinc and Castor oil Cream					

For peer review only

For peer review only - http://bmjopen.bmj.com/site/about/guidelines.xhtml

Table S2: Top five emollients recommended by type (n=102)

Lotion			Cream			Gel			Ointment		
Name	n	%	Name	n	%	Name	n	%	Name	n	%
Dermol 500 Lotion	71	70	Cetraben Cream	70	69	Doublebase Gel	62	61	White soft/Liquid paraffin 50/50	81	79
E45 Lotion	31	30	Zerobase Cream	62	61	Zerodouble Gel	39	38	Emulsifying Ointment BP	80	78
Cetraben Lotion	30	29	Dermol Cream	60	59	Doublebase Dayleve Gel	21	21	Hydromol Ointment	72	71
Aveeno Lotion	28	27	Zerocream	56	55	Oilatum Gel	8	8	Zeroderm Ointment	59	58
Eucerin Intensive Lotion	22	22	Balneum plus cream	53	52	Isomol Gel	6	6	Paraffin White Soft	41	40

For peer review only - http://bmjopen.bmj.com/site/about/guidelines.xhtml

Table S3: Top five recommended emollients by order of formulary preference (n=102 formularies)

Emollient	Number (%) of formularies recommending as				Recommended without ranking
	First line	Second line	Third line	Fourth line	
Emulsifying Ointment BP	31 (30%)	8 (8%)	0 (0%)	0 (0%)	41 (40%)
Zeroderm Ointment	28 (27%)	11 (11%)	1 (1%)	1 (1%)	18 (18%)
Zerobase Cream	27 (26%)	8 (8%)	2 (2%)	0 (0%)	25 (25%)
Zerocream	25 (25%)	5 (5%)	1 (1%)	0 (0%)	25 (25%)
White soft/ Liquid paraffin 50/50	23 (23%)	9 (9%)	2 (2%)	0 (0%)	48 (47%)

For peer review only

For peer review only - http://bmjopen.bmj.com/site/about/guidelines.xhtml

STROBE 2007 (v4) Statement—Checklist of items that should be included in reports of *cross-sectional studies*

Section/Topic	Item #	Recommendation	Reported on page #
**Title and abstract**	1	(*a*) Indicate the study's design with a commonly used term in the title or the abstract	1
		(*b*) Provide in the abstract an informative and balanced summary of what was done and what was found	2
**Introduction**			
Background/rationale	2	Explain the scientific background and rationale for the investigation being reported	3
Objectives	3	State specific objectives, including any prespecified hypotheses	2 & 3
**Methods**			
Study design	4	Present key elements of study design early in the paper	3
Setting	5	Describe the setting, locations, and relevant dates, including periods of recruitment, exposure, follow-up, and data collection	3
Participants	6	(*a*) Give the eligibility criteria, and the sources and methods of selection ~~of participants~~	3
Variables	7	Clearly define all outcomes, ~~exposures, predictors, potential confounders, and effect modifiers. Give diagnostic criteria, if applicable~~	2 & 3
Data sources/ measurement	8*	For each variable of interest, give sources of data and details of methods of assessment (measurement). ~~Describe comparability of assessment methods if there is more than one group~~	3
Bias	9	Describe any efforts to address potential sources of bias	*3*
Study size	10	Explain how the study size was arrived at	N/A
Quantitative variables	11	Explain how quantitative variables were handled in the analyses. If applicable, describe which groupings were chosen and why	3
Statistical methods	12	(*a*) Describe all statistical methods, including those used to control for confounding	3
		(*b*) Describe any methods used to examine subgroups and interactions	N/A
		(*c*) Explain how missing data were addressed	3 & 6
		(*d*) If applicable, describe analytical methods taking account of sampling strategy	N/A
		(*e*) Describe any sensitivity analyses	N/A
**Results**			

For peer review only

Participants	13*	(a) Report numbers of individuals at each stage of study—eg numbers potentially eligible, examined for eligibility, confirmed eligible, included in the study, completing follow-up, and analysed	3 & 12
		(b) Give reasons for non-participation at each stage	6
		(c) Consider use of a flow diagram	12
Descriptive data	14*	(a) Give characteristics of study participants (eg demographic, clinical, social) and information on exposures and potential confounders	N/A
		(b) Indicate number of participants with missing data for each variable of interest	N/A
Outcome data	15*	Report numbers of outcome events or summary measures	3, 4, 5
Main results	16	(*a*) Give unadjusted estimates and, if applicable, confounder-adjusted estimates and their precision (eg, 95% confidence interval). Make clear which confounders were adjusted for and why they were included	3, 4, 5
		(*b*) Report category boundaries when continuous variables were categorized	N/A
		(*c*) If relevant, consider translating estimates of relative risk into absolute risk for a meaningful time period	N/A
Other analyses	17	Report other analyses done—eg analyses of subgroups and interactions, and sensitivity analyses	N/A
**Discussion**			
Key results	18	Summarise key results with reference to study objectives	5
Limitations	19	Discuss limitations of the study, taking into account sources of potential bias or imprecision. Discuss both direction and magnitude of any potential bias	2 & 5
Interpretation	20	Give a cautious overall interpretation of results considering objectives, limitations, multiplicity of analyses, results from similar studies, and other relevant evidence	5 & 6
Generalisability	21	Discuss the generalisability (external validity) of the study results	5 & 6
**Other information**			
Funding	22	Give the source of funding and the role of the funders for the present study and, if applicable, for the original study on which the present article is based	8

*Give information separately for cases and controls in case-control studies and, if applicable, for exposed and unexposed groups in cohort and cross-sectional studies.

Note: An Explanation and Elaboration article discusses each checklist item and gives methodological background and published examples of transparent reporting. The STROBE checklist is best used in conjunction with this article (freely available on the Web sites of PLoS Medicine at http://www.plosmedicine.org/, Annals of Internal Medicine at http://www.annals.org/, and Epidemiology at http://www.epidem.com/). Information on the STROBE Initiative is available at www.strobe-statement.org.

For peer review only
